# Human 2′-Deoxynucleoside 5′-Phosphate *N*-Hydrolase 1: Mechanism of 2′-Deoxyuridine 5′-Monophosphate Hydrolysis

**DOI:** 10.1021/acs.biochem.3c00369

**Published:** 2023-08-15

**Authors:** Suneeta Devi, Anna E. Carberry, Greice M. Zickuhr, Alison L. Dickson, David J. Harrison, Rafael G. da Silva

**Affiliations:** † School of Biology, Biomedical Sciences Research Complex, University of St Andrews, St Andrews KY16 9ST, U.K.; ‡ School of Medicine, University of St Andrews, St Andrews KY16 9TF, U.K.; § NuCana Plc, Edinburgh EH12 9DT, U.K.

## Abstract

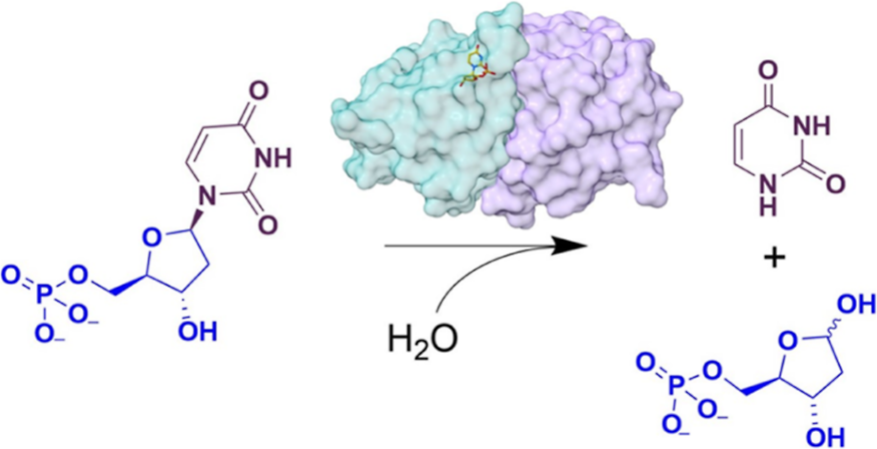

The enzyme 2′-deoxynucleoside 5′-phosphate *N*-hydrolase 1 (DNPH1) catalyzes the *N*-ribosidic bond cleavage of 5-hydroxymethyl-2′-deoxyuridine 5′-monophosphate to generate 2-deoxyribose 5-phosphate and 5-hydroxymethyluracil. DNPH1 accepts other 2′-deoxynucleoside 5′-monophosphates as slow-reacting substrates. DNPH1 inhibition is a promising strategy to overcome resistance to and potentiate anticancer poly(ADP-ribose) polymerase inhibitors. We solved the crystal structure of unliganded human DNPH1 and took advantage of the slow reactivity of 2′-deoxyuridine 5′-monophosphate (dUMP) as a substrate to obtain a crystal structure of the DNPH1:dUMP Michaelis complex. In both structures, the carboxylate group of the catalytic Glu residue, proposed to act as a nucleophile in covalent catalysis, forms an apparent low-barrier hydrogen bond with the hydroxyl group of a conserved Tyr residue. The crystal structures are supported by functional data, with liquid chromatography–mass spectrometry analysis showing that DNPH1 incubation with dUMP leads to slow yet complete hydrolysis of the substrate. A direct UV-vis absorbance-based assay allowed characterization of DNPH1 kinetics at low dUMP concentrations. A bell-shaped pH-rate profile indicated that acid–base catalysis is operational and that for maximum *k*_cat_/*K*_M_, two groups with an average p*K*_a_ of 6.4 must be deprotonated, while two groups with an average p*K*_a_ of 8.2 must be protonated. A modestly inverse solvent viscosity effect rules out diffusional processes involved in dUMP binding to and possibly uracil release from the enzyme as rate limiting to *k*_cat_/*K*_M_. Solvent deuterium isotope effects on *k*_cat_/*K*_M_ and *k*_cat_ were inverse and unity, respectively. A reaction mechanism for dUMP hydrolysis is proposed.

The enzyme 2′-deoxynucleoside 5′-phosphate *N*-hydrolase 1 (DNPH1) (EC 3.2.2.-) is responsible for catabolizing toxic 5-hydroxymethyl-2′-deoxyuridine 5′-monophosphate (5hmdUMP), a noncanonical 2′-deoxynucleotide that, if incorporated into DNA, must be excised via DNA repair pathways.^1^ Breast cancer gene (BRCA)-deficient cancers such as breast, pancreatic, prostate, and ovarian cancers are vulnerable to treatment with poly(ADP-ribose) polymerase inhibitors (PARPi) as they rely on poly(ADP-ribose) polymerase for essential DNA repair to support rapid proliferation because they cannot carry out homologous recombination.^1−3^ This inhibition is highly dependent on accumulation of 5hmdUMP, which is erroneously incorporated into DNA causing poly(ADP-ribose) polymerase to be recruited to the repair site, where it is trapped by PARPi.^4^ However, resistance to PARPi arises in cancer cells due to upregulation of DNPH1 and the consequent depletion of 5hmdUMP; thus, inhibition of DNPH1 has been demonstrated to be an attractive strategy to potentiate PARPi and resensitize cancer cells to their action.^1^

Although 5hmdUMP is the physiological substrate of *Homo sapiens* DNPH1^1^ (*Hs*DNPH1), the enzymatic activity was originally discovered with the rat orthologue acting on canonical 2′-deoxynucleoside 5′-monophosphates. DNPH1 catalyzes the hydrolysis of the *N*-ribosidic bond of 2′-deoxynucleoside 5′-monophosphates to produce 2-deoxyribose 5-phosphate and the corresponding nucleobase (Scheme 1).^5^*Hs*DNPH1 also accepts canonical 2′-deoxynucleoside 5′-monophosphates as substrates, albeit with very low reaction rates.^6^ Crystal structures of the rat orthologue have been reported in complex with N6-substituted AMP analogues,^6,7^ and a crystal structure of the human orthologue was solved in complex with N6-naphthyl-AMP.^8^ All those N6-substituted AMP analogues harbored a 2′-hydroxyl group and are not DNPH1 substrates.^5−8^ Based on the amino acid sequence and tertiary structure, DNPH1 is related to nucleoside 2′-deoxyribosyltransferases (EC 2.4.2.6),^5,7^ which catalyze the reversible transfer of a 2-deoxyribosyl group between two nucleobases by a double-displacement mechanism via a covalent 2-deoxyribosyl-enzyme intermediate where an active-site Glu residue acts as a nucleophile.^9^

**Scheme 1:**
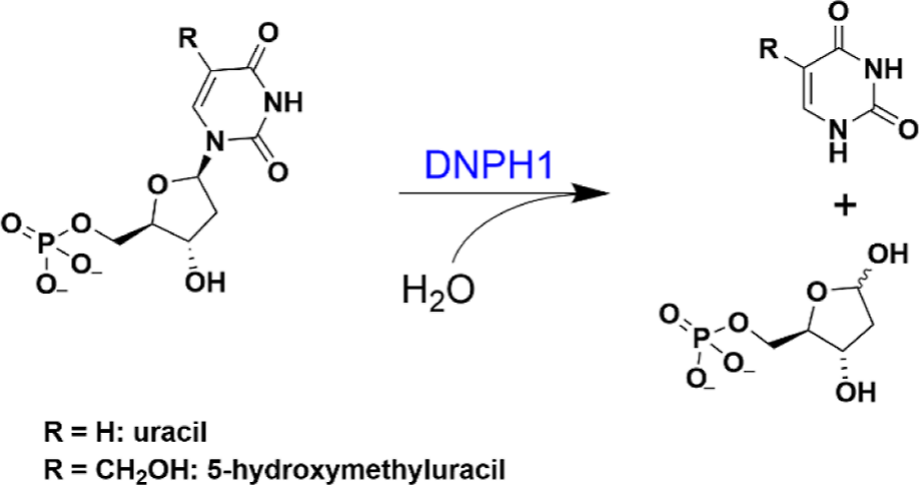
DNPH1-Catalyzed Reaction Hydrolysis of either 5hmdUMP or dUMP is shown as an example.

Although direct evidence for a covalent intermediate in DNPH1 catalysis is still elusive, its catalytic mechanism has been proposed to involve such an intermediate.^10^ Evidence for this proposal includes the conservation of key active-site residues in DNPH1 and nucleoside 2′-deoxyribosyltransferases, such as Tyr24, Asp80, and Glu104 (*Hs*DNPH1 numbering),^5,7^ the catalytic essentiality of the putatively nucleophilic Glu residue in the rat and human DNPH1 orthologues,^1,10^ and the retention of stereochemical configuration on the anomeric carbon upon rat DNPH1-catalyzed methanolysis of 2′-deoxyguanosine 5′-monophosphate.^10^

Here, we used protein crystallography, liquid chromatography-electrospray ionization-mass spectrometry (LC-ESI-MS), differential scanning fluorimetry (DSF), high-performance liquid chromatography (HPLC), pH-rate profiles, solvent viscosity effects, and solvent deuterium isotope effects to characterize the *Hs*DNPH1-catalyzed hydrolysis of 2′-deoxyuridine 5′-monophosphate (dUMP). Our results provide a detailed view of the Michaelis complex and shed light on rate-limiting steps, which may inform future inhibitor design targeting this enzyme.

## Materials and Methods

### Materials

All commercially available chemicals were used without further purification. BaseMuncher endonuclease was purchased from AbCam. Ampicillin, dithiothreitol (DTT), and isopropyl-β-D-1-thiogalactopyranoside (IPTG) were purchased from Formedium. *Escherichia coli* DH5α (high efficiency) and BL21(DE3) cells and Gibson Assembly Cloning Kit were purchased from New England Biolabs. Ethylenediaminetetraacetic acid (EDTA)-free Complete protease inhibitor cocktail was purchased from Roche. Deuterium oxide (D_2_O), sodium deuteroxide, 2-(*N*-morpholino)ethanesulfonic acid (MES), *N*-[tris(hydroxymethyl)methyl]-3-aminopropanesulfonic acid (TAPS), glycerol, imidazole, lysozyme, NiCl_2_, tris(hydroxymethyl)aminomethane (Tris), polyethylene glycol 8000 (PEG-8000), and dUMP were purchased from Merck. Agarose, dNTPs, 4-(2-hydroxyethyl)piperazine-1-ethanesulfonic acid (HEPES), NaCl, and Phusion high-fidelity polymerase were purchased from ThermoFisher Scientific. Tobacco etch virus protease (TEVP) was produced as previously described.^11^

### Expression of *Hs*DNPH1 and Truncated *Hs*DNPH1

The DNA-encoding *Hs*DNPH1 (UniProt O43598) and a truncated *Hs*DNPH1 (*Hs*DNPH1^Trunc^), each containing a TEVP-cleavable N-terminal His-tag with restriction sites for NdeI and HindIII at the 5′- and 3′-ends, respectively, were purchased as codon-optimized (for expression in *E. coli*) gBlocks (IDT). *Hs*DNPH1- and *Hs*DNPH1^Trunc^-encoding gBlocks were polymerase chain reaction-modified and inserted into modified pJexpress414 plasmids using Gibson Assembly^12^ according to the manufacturer’s instructions (New England Biolabs). Constructs were used to transform *E. coli* DH5α-competent cells (New England Biolabs), were subsequently sequenced (Eurofins) to confirm the insertion of the gene and that no mutations had been introduced, and then used to transform *E. coli* BL21(DE3)-competent cells (New England Biolabs). Transformed cells were grown independently in lysogeny broth containing 100 μg mL^–1^ ampicillin at 37 °C until an optical density at 600 nm (OD_600_) of 0.6–0.8 before expression was induced with 0.5 mM IPTG. Cells were grown for an additional 3 h, harvested by centrifugation (6774*g*, 15 min, 4 °C), and stored at −20 °C.

### Purification of *Hs*DNPH1 and *Hs*DNPH1^Trunc^

Each recombinant protein, initially encoded as MHHHHHHENLYFQG-protein sequence (Met–6×His-tag–TEVP cleavage site–protein sequence), was purified independently, and all purification procedures were conducted on ice or at 4 °C using an ÄTKA Start FPLC system (GE Healthcare). All filtration of samples was by a syringe through a 0.45 μm membrane. All sodium dodecyl sulfate-polyacrylamide gel electrophoresis (SDS-PAGE) used a NuPAGE Bis-Tris 4–12% Precast Gel (ThermoFisher Scientific) with the PageRuler Plus Stained Protein Ladder (ThermoFisher Scientific). Proteins were concentrated through a 10 000 molecular weight cutoff (MWCO) ultrafiltration membrane (Millipore). Cells were thawed on ice and resuspended in Buffer A [30 mM Tris–HCl, 150 mM NaCl, and 10 mM imidazole pH 7.5] containing lysozyme (0.2 mg mL^–1^), BaseMuncher (Expedeon) (0.05 mg mL^–1^), and half a tablet of EDTA-free Complete protease inhibitor cocktail, disrupted in a cell disruptor (Constant systems) at 30 kpsi and centrifuged (48 000*g*, 30 min). The supernatant was filtered and loaded onto a 5 mL HisTrap FF column (Cytiva) pre-equilibrated with Buffer A. The column was washed with 10 column volumes (CV) of either Buffer A. The desired protein was eluted with a 20-CV linear gradient of 0–90% Buffer B [30 mM Tris–HCl, 150 mM NaCl, and 500 mM imidazole pH 7.5]. Fractions containing the protein of interest were pooled, mixed with TEVP at a ratio of 15 mg of target protein to 1 mg of TEVP, and dialyzed against 2 × 2 L Buffer C [20 mM HEPES, 150 mM NaCl, 2 mM DTT, 10% glycerol pH 7.5] over a 48 h period and then dialyzed against 2 × 2 L of Buffer A over a period of 24 h. Samples were filtered and loaded onto a 5 mL HisTrap FF column pre-equilibrated with Buffer A. The flow through was collected and analyzed by SDS-PAGE. Each purified protein was dialyzed against 2 × 2 L of 20 mM HEPES pH 8.0. Proteins were concentrated, and their concentrations were determined by UV absorbance (NanoDrop) at 280 nm using theoretical molar extinction coefficients (ε_280_) of 23 950 M^–1^ cm^–1^ (*Hs*DNPH1) and 18 450 M^–1^ cm^–1^ (*Hs*DNPH1^Trunc^) (ProtParam tool—Expasy). Proteins were aliquoted and stored at −80 °C. Their molecular masses were determined by LC-ESI-MS analysis.

### Protein Crystallography

*Hs*DNPH1^Trunc^ was concentrated to 8 mg mL^–1^ in 30 mM Tris–HCl pH 7.5, 150 mM NaCl, and crystallized at 20 °C using the hanging drop method in 24-well plates by mixing 2.5 μL of protein with 2.5 μL of reservoir solution consisting of 40 mM sodium propionate, 20 mM MES, and 40 mM Bis-Tris-propane pH 4.0 and 20% PEG 1500. Each well of the 24-well plate contained 1 mL of reservoir solution. Crystals were flash-frozen in liquid nitrogen with a cryoprotectant (5% PEG-1500 and 35% PEG-400 in the reservoir buffer). To obtain the dUMP-bound crystals, crystals were soaked in the cryoprotectant supplemented with 50 mM of dUMP for less than 1 min before flash-freezing. X-ray diffraction data were collected at beamline I04 at Diamond Light Source, UK (wavelength 0.9537 Å). Unliganded *Hs*DNPH1^Trunc^ data were processed and scaled using the automated processing pipeline autoPROC. For the *Hs*DNPH1^Trunc^:dUMP complex, unmerged MTZ was processed and scaled using the automated processing pipeline Xia2^13^ integrated with DIALS^14^ and further processed with AIMLESS.^15^ Unliganded *Hs*DNPH1^Trunc^ structures were solved by molecular replacement with PhaserMR^16^ using the inhibitor-bound human DNPH1 structure (PDB ID: 4P5E)^8^ as the search model, whereas dUMP-bound structures were solved with the unliganded structure as the search model. Structures were refined using iterative cycles of model building and refinement with COOT^17^ and Phenix.refine,^18^ respectively. The final structures were rerefined using PDB-REDO.^19^ In the unbound structure, whose asymmetric unit shows four subunits, no electron density was visible for Arg30–Glu34 (all subunits), Val57–Glu68 (two of the subunits), and Glu55–Ala70 (two of the subunits). In the dUMP-bound structure, whose asymmetric unit has two subunits, no electron density was visible for Glu55–Ala70 (both subunits) and Ile29–Glu34 (one of the subunits). These residues were therefore not modeled. Coordinates and structure factor files have been deposited in the Protein Data Bank (PDB IDs 8OS9 and 8OSC).

### DSF-Based Thermal Denaturation of *Hs*DNPH1

DSF measurements (λ_ex_ = 490 nm and λ_em_ = 610 nm) were performed in 96-well plates on a Stratagene Mx3005p instrument. Reactions (50 μL) contained 6 μM *Hs*DNPH1 in 50 mM HEPES pH 7.5 and 150 mM NaCl. Invitrogen Sypro Orange (5×) was added to all wells. Controls lacked protein and were subtracted from the corresponding protein-containing samples. Thermal denaturation curves were recorded over a temperature range of 25–93 °C with increments of 1 °C min^–1^. Three independent measurements were carried out.

### Analysis of the *Hs*DNPH1 Reaction by LC-ESI-MS

A reaction mixture (200 μL) containing 100 mM HEPES pH 7.5, 40 μM *Hs*DNPH1, and 40 μM dUMP was incubated at 37 °C for 2 h. The control lacked enzymes. After 2 h, 400 μL of ice-cold methanol was added, and the sample was vortexed for 5 min and centrifuged for 10 min (16 000*g*). The supernatant was collected and dried under a stream of N_2_ gas, and resolubilized in 200 μL of HPLC-grade water. Reaction and control samples (1 μL from each) were loaded onto an ACE Excel 2 AQ column (2.1 × 100 mm) on a Waters ACQUITY UPLC system coupled to a Xevo G2-XS QToF mass spectrometer equipped with an ESI source. The UPLC mobile phase was (A) 0.1% formic acid in water and (B) 0.1% formic acid in acetonitrile. Samples were chromatographed over the following protocol: 0–1 min at 99% (A) and 1% (B); 1–3 min linear gradient from 99% (A) and 1% (B) to 1% (A) and 99% (B); and 3–3.5 min at 1% (A) and 99% (B), a flow rate of 0.2 mL min^–1^, a column temperature of 40 °C. ESI data were acquired in negative mode with a capillary voltage of 2500 V. The source and desolvation gas temperatures of the mass spectrometer were 100 and 250 °C, respectively. The cone gas flow was 50 L h^–1^, while the gas flow was 600 L h^–1^. A scan was performed between 50–1200 *m*/*z*, and masses for dUMP, uracil, and 2-deoxyribose 5-phosphate were extracted in turn. A lockspray signal was measured, and a mass correction was applied by collecting every 10 s, averaging 3 scans of 0.5 s each, using Leucine Enkephalin as a correction factor for mass accuracy.

### HPLC-Based Assay of *Hs*DNPH1 Activity

Reactions (50 μL) containing 100 mM HEPES pH 7.5, 20 μM *Hs*DNPH1, and varying concentrations of dUMP (0–32 mM) were run for 15 min at 37 °C, quenched with 200 μL of ice-cold methanol, vortexed for 3 min, and centrifuged (16 000*g*) for 6 min. The supernatant was collected, dried under vacuum centrifugation, and resolubilized in HPLC-grade water before 10 μL was loaded onto an Atlantis Premier BEH C18 AX column (1.7 μm, 2.1 × 100 mm) on a Thermo Dionex UltiMate 3000 HPLC. The column was pre-equilibrated with 50 mM triethylamine:acetic acid pH 5.0 and run with the same mobile phase in an isocratic manner at 0.35 mL min^–1^. Elution was monitored by UV absorbance at 260 nm. Controls lacked enzyme. The exact same procedure was carried out with reactions run in 90% D_2_O (v/v), where pD = pH meter reading + 0.4.^20^ A uracil and dUMP standard mixture was treated according to the reaction conditions (without an enzyme) and run to determine respective retention times. A uracil standard curve was obtained by injecting uracil samples of known concentrations (0.05–0.8 mM) treated exactly the same as the reactions. Reaction product quantification was carried out by integrating the uracil peaks and using the standard curve. All experiments were carried out twice independently. Reaction progression at 15 min was less than 10% in all cases, and product formation per min was assumed to represent initial rates.

### Spectrophotometric Assay of *Hs*DNPH1 Activity

Initial rates of enzyme-catalyzed hydrolysis of dUMP at 37 °C were monitored continuously for 10 min in 1 cm optical path length quartz cuvettes (Hellma) following the decrease in absorbance at 282 nm (Δε_282_ = 1600 M^–1^ cm^–1^)^21^ in a Shimadzu UV-2600 spectrophotometer outfitted with a CPS unit for temperature control. Reactions (500 μL) contained 100 mM HEPES pH 7.5, 20 μM either *Hs*DNPH1 or *Hs*DNPH1^Trunc^ and varying concentrations of dUMP. The maximum dUMP concentration never exceeded 700 μM as the linear range of the spectrophotometer at 282 nm was extrapolated at higher concentrations. Controls lacked enzymes.

### *Hs*DNPH1 pH-Rate Profile

The pH dependence of the reaction rate constant at very low substrate concentration (*k*_cat_/*K*_M_) was determined by measuring initial rates at 37 °C via the spectrophotometric assay in a composite buffer system of 100 mM MES, 100 mM HEPES, and 100 mM TAPS pH 6.0–8.7 (all pH values determined at 37 °C) using either 20 μM *Hs*DNPH1 (pH 6.0–8.5) or 30 μM *Hs*DNPH1 (pH 8.7) and varying dUMP concentrations (pH 6.5–8.0: 0–400 μM dUMP; pH 6.0, 8.5, and 8.7: 0–700 μM dUMP). To ensure enzyme stability at the extremes of the pH range, *Hs*DNPH1 was diluted in buffer at either pH 6.0 or 8.7 prior to the activity assay at pH 7.5, without any change in activity within experimental error. Three independent measurements were carried out.

### *Hs*DNPH1 Activity in D_2_O and Glycerol

Initial rates of dUMP hydrolysis were measured at 37 °C via the spectrophotometric assay in 100 mM HEPES pH 7.5, 20 μM *Hs*DNPH1 and varying concentrations of dUMP (0–400 μM) in the presence of 0–18% glycerol (v/v) or in the presence of 5% PEG-8000 (v/v) (as a macroviscogen control). Alternatively, initial rates of dUMP hydrolysis were measured in 100 mM HEPES pL 7.5 (pD = pH meter reading + 0.4),^20^ 20 μM *Hs*DNPH1, and varying concentrations of dUMP (0–400 μM) in either H_2_O or 95.5% D_2_O (v/v). Three independent measurements were carried out.

### Nuclear Magnetic Resonance Analysis of the Reaction

Reactions (600 μL) in 95.5% D_2_O (v/v) contained 100 mM HEPES pD 7.5, 40 μM *Hs*DNPH1, and 10 mM dUMP in the presence or absence of 18% glycerol (v/v). Controls lacked enzymes. Reactions and controls were incubated for 4 h at 37 °C, after which the enzyme was removed by filtration through 10 000-MWCO Vivaspin centrifugal concentrators. Standards with either 10 mM of uracil or 2-deoxyribose 1-phosphate were prepared in 100 mM HEPES pD 7.5 in D_2_O. Each ^1^H nuclear magnetic resonance (NMR) spectrum was acquired in a Bruker AVIII 500 MHz in a total of four scans with 4 s of acquisition time per scan. Solvent lock was achieved with deuterium.

### Analysis of Kinetic Data

Kinetics data were analyzed by the nonlinear regression function of SigmaPlot 14.0 (SPSS Inc.). Data points and error bars represent either mean ± SEM or mean ± SD, and kinetic and equilibrium constants are given as mean ± fitting error. Equations 1–5 were fitted to, respectively, data for initial-rate dependence on substrate concentration at very low dUMP concentrations, pH-rate profile, solvent viscosity effect, substrate saturation curves, and solvent deuterium kinetic isotope effect from the HPLC-based assay (the latter was also calculated as the ratio of the relevant rate constant in H_2_O and D_2_O). In equations 1–5, *v* is the initial rate, *k*_cat_ is the steady-state turnover number, *K*_M_ is the Michaelis constant, *E*_T_ is total enzyme concentration, *S* is the concentration of substrate, (*k*_cat_/*K*_M_)_0_ and (*k*_cat_/*K*_M_)_η_ are the rate constants in the absence and presence of glycerol, respectively, η_rel_ is the relative viscosity of the solution, *A* and *B* are parameters required to describe the hyperbolic behavior,^22^*F*_i_ is the fraction of deuterium label,  is the solvent isotope effect minus 1 on , *C* is the pH-independent value of *k*_cat_/*K*_M_, *H* is the proton concentration, and *K*_a1_ and *K*_a2_ are apparent acid dissociation constants.
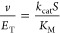
1
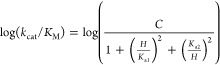
2
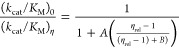
3
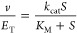
4
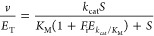
5

## Results and Discussion

### Purification and Biophysical Characterization of *Hs*DNPH1 and *Hs*DNPH1^Trunc^

*Hs*DNPH1 was purified to homogeneity as estimated by Coomassie Blue-stained SDS-PAGE (Figure S1A), and LC-ESI-MS analysis confirmed that the mass of the purified protein (19 164.9) (Figure S1B) matched the expected value of 19 165.5 based on the amino acid sequence (Figure S2). Thermal denaturation analysis by DSF (Figure S3) was fitted to a Boltzmann equation^23^ and yielded a melting temperature of 61.5 ± 0.1 °C. *Hs*DNPH1^Trunc^ was purified to homogeneity as evidenced by Coomassie Blue-stained SDS-PAGE (Figure S4A), and LC-ESI-MS analysis confirmed that the mass of the purified protein (16 173.6) (Figure S4B) was in agreement with the expected value of 16 174.2 based on the amino acid sequence (Figure S2). This truncated version was produced because attempts to crystallize full-length *Hs*DNPH1 were unsuccessful, and previous structural work adopted this strategy to obtain crystals of the rat orthologue^6,7^ and of the human orthologue bound to an inhibitor.^8^

### Crystal Structures of Unbound and dUMP-Bound *Hs*DNPH1

**Figure 1:**
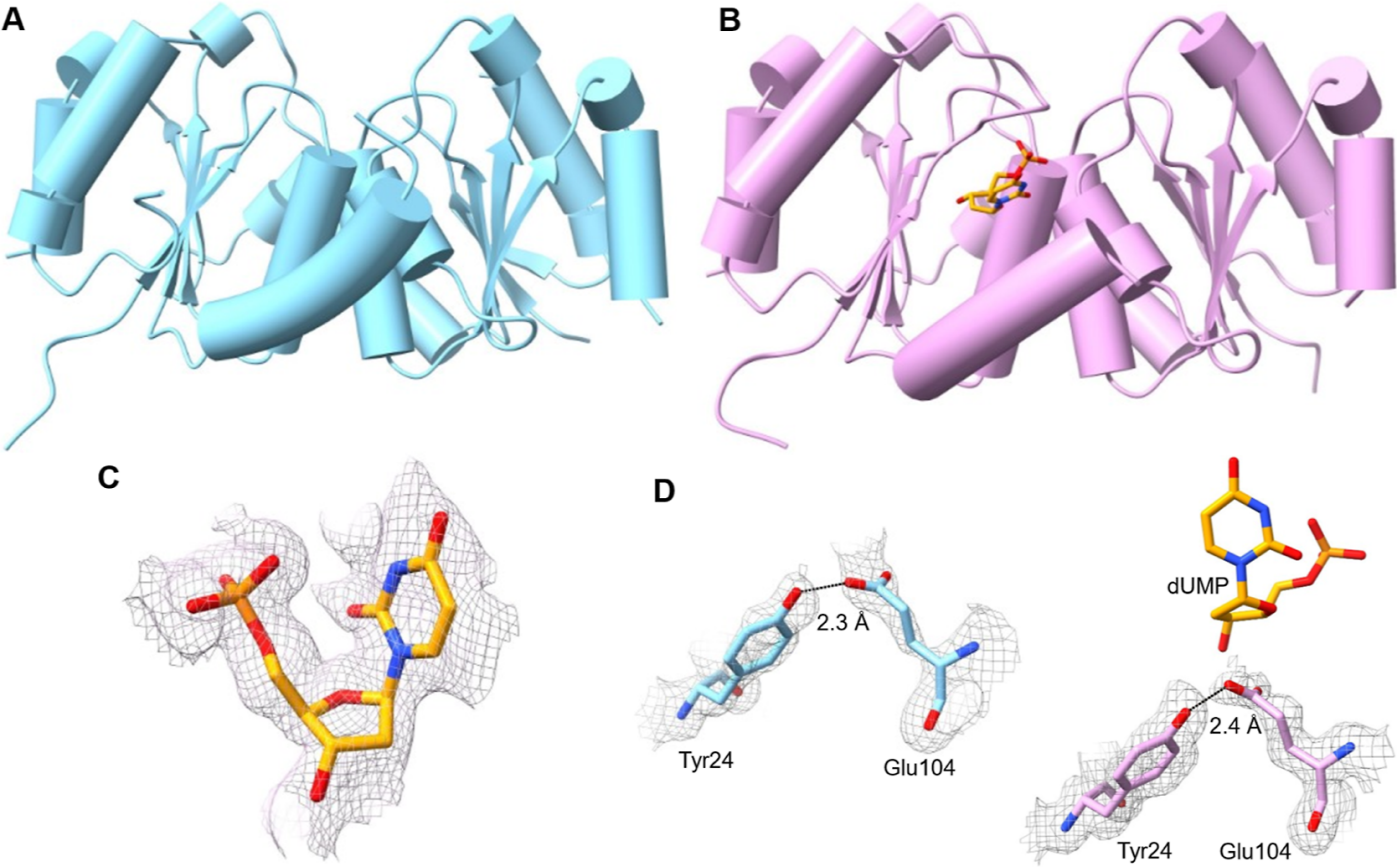
*Hs*DNPH1 crystal structures. (A) Cartoon representation of the unliganded *Hs*DNPH1^Trunc^ dimer. (B) Cartoon representation of the *Hs*DNPH1^Trunc^:dUMP complex dimer. The ligand is depicted as a stick model, with carbon in yellow, oxygen in red, nitrogen in blue, and phosphorus in orange. Only one of the active sites is occupied by the ligand. (C) Omit (*F*_obs_–*F*_calc_) electron density map of the bound dUMP contoured at 1.0 σ. (D) Apparent LBHB (dashed line) between Tyr24 and Glu104 in unliganded *Hs*DNPH1^Trunc^ (left) and *Hs*DNPH1^Trunc^:dUMP complexes (right). The electron density maps (2*F*_obs_–*F*_calc_) of the interacting residues are contoured at 1.0 σ.

To investigate the structural basis for catalysis of *N*-ribosidic bond cleavage, the structures of unbound *Hs*DNPH1^Trunc^ and dUMP-bound complex were solved with data to 1.7 and 1.42 Å resolution, respectively. Data processing and refinement statistics are summarized in Table S1. Unliganded *Hs*DNPH1^Trunc^ and dUMP complex structures have four and two molecules in the asymmetric unit, comprising two dimers and one dimer, respectively. Buried surface areas were calculated by PISA^24^ analysis to be 1241.0 and 1267.5 Å^2^ for unbound and substrate-bound dimers, respectively. The dimeric form is common for DNPH1 and is essential for catalysis.^10,25^ The overall fold is similar to previously reported inhibitor-bound structures of *Hs*DNPH1 and rat DNPH1,^6−8^ with a five-stranded parallel β-sheet core surrounded by five α-helices (Figure 1A,B). Omit (*F*_obs_–*F*_calc_) maps showed electron density for dUMP (Figure 1C) but only in half of the binding sites of the dimer (Figure 1B). Attempts to elucidate if this observation had implications for potential negative cooperativity in substrate binding using isothermal titration calorimetry failed to detect any binding of UMP (expected to act as an inhibitor) to *Hs*DNPH1 beyond the heat of dilution, which might be a consequence of the very high *K*_M_ reported for dUMP (assuming UMP would bind with similar weak affinity).^6^ Binding of dUMP stabilizes the Ile29–Glu34 loop, for which no electron density can be seen in the absence of the substrate, probably as a result of high flexibility. Overlay of the unliganded and Michaelis complex structures (Figure S5A) yielded a small root-mean-square deviation of 0.52 Å over 117 Cα atoms.

An intriguing feature of the *Hs*DNPH1^Trunc^ crystal structures is the presence of an apparent low-barrier hydrogen bond (LBHB) between the −COO^–^ and −OH groups of the highly conserved residues Glu104 and Tyr24 as inferred from the O–O distances in unbound *Hs*DNPH1^Trunc^ and *Hs*DNPH1^Trunc^:dUMP complexes, respectively (Figure 1D). LBHBs may form when the p*K*_a_s of the hydrogen bond (H-bond) donor and acceptor groups are closely matched, and the distance between the two electronegative atoms sharing the H-bond is shorter than the sum of their van der Waals radii, *i.e.*, shorter than 2.55 Å for two O atoms.^26^ The distance between the O atoms of the Glu104 −COO^–^ and Tyr24 −OH groups is 2.3 Å in the unbound and 2.4 Å in the dUMP-bound enzyme (Figure 1D). As the Glu residue in the equivalent position to Glu104 is proposed to act as a nucleophile in the rat DNPH1,^10^ the apparent LBHB might be involved in tuning the position and nucleophilicity of Glu104 in *Hs*DNPH1.

### *Hs*DNPH1 Interaction with dUMP

**Figure 2:**
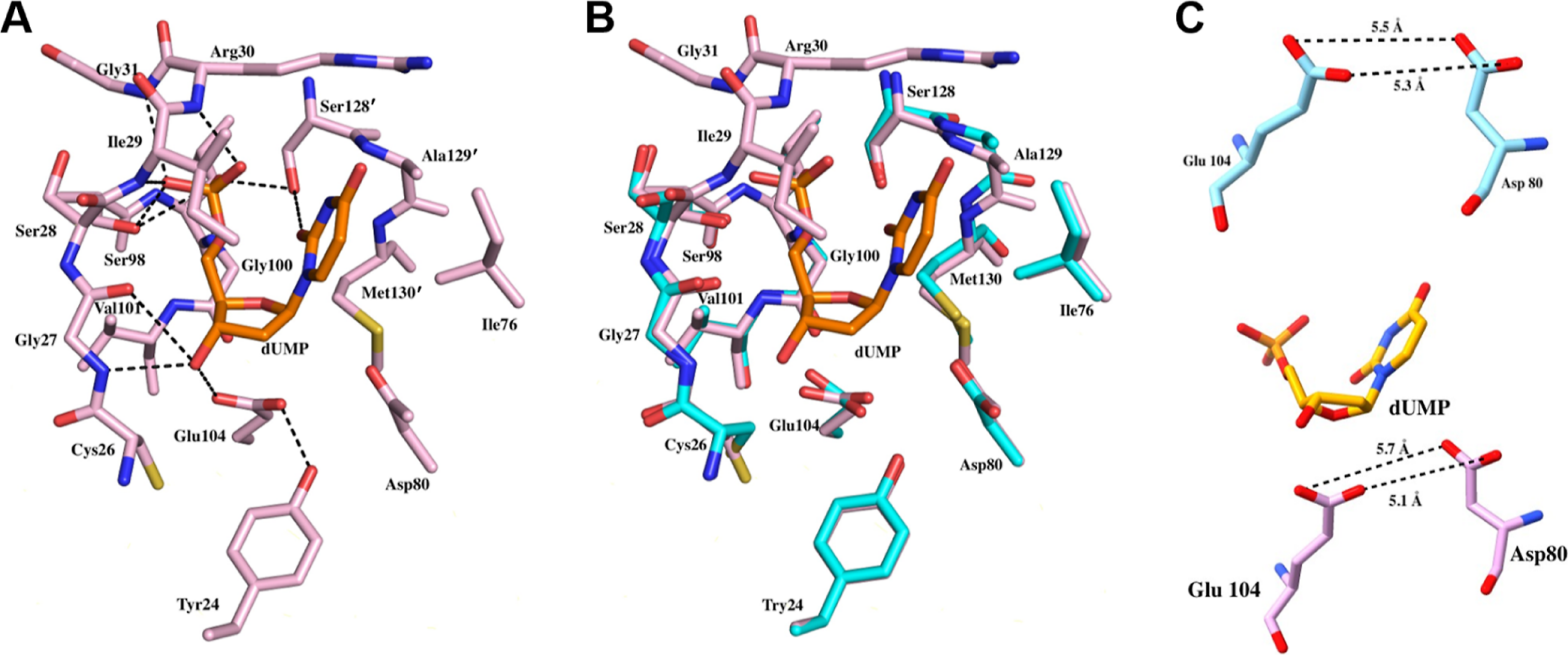
*Hs*DNPH1 active site. (A) Stick model of *Hs*DNPH1^Trunc^:dUMP complex active site. Carbon atoms are shown in pink in the protein and yellow in the substrate. Ser128′, Ala129′, and Met130′ are contributed by the adjacent subunit. Dashed lines depict polar interactions with dUMP. (B) Overlay of the *Hs*DNPH1^Trunc^:dUMP complex (pink) and unbound *Hs*DNPH1^Trunc^ (cyan). (C) Distance between the carboxylate groups of Glu104 and Asp80 in the unbound (top) and dUMP-bound enzyme (bottom). Dashed lines in this case depict distances, not interactions.

The active site of *Hs*DNPH1 encompasses residues from both subunits (Figure 2A). The 5′-PO_4_^2–^ group of dUMP makes polar contacts with the main chain −NH of Ile29, Arg30, Gly31, and Gly100, the Ser98 −OH of one subunit, and the Ser128 −OH of the adjacent subunit. The dUMP 3′-OH donates an H-bond to one of the O atoms of Glu104 −COO^–^ and accepts an H-bond from Gly27 −NH. The other O atom of Glu104 −COO^–^ is locked in an apparent LBHB with the Tyr24 −OH. The uracil moiety of dUMP makes nonpolar contact with Ile76 of one subunit, and its 2-CO group makes a polar interaction with the −OH of Ser128 of the adjacent subunit. Additional nonpolar interactions involve the nucleobase and Ala129 and Met130 of the adjacent subunit. Glu104 −COO^–^ sits underneath the 2′-deoxyribose moiety of dUMP, 3.8 Å away from the dUMP C1′, a position suitable for nucleophilic attack on the anomeric carbon of the substrate (Figure 2A). It is therefore surprising that the intact substrate was trapped in the active site. The main change reflected in the active site upon dUMP binding is the stabilization of the Ile29–Glu34 loop via polar interactions with the 5′-PO_4_^2–^ group, with the side chain of Arg30 closing as a lid over the substrate (Figure 2B). An intriguing hypothesis for this observation invokes the enzyme harnessing the binding energy for the 5′-PO_4_^2–^ group to drive a conformational change that traps the substrate in a caged Michaelis complex conducive to catalysis.^27^ This would help explain the strict requirement for a 5′-PO_4_^2–^ group for DNPH1 catalysis and the enzyme’s inability to accept 2′-deoxynucleosides as substrates.^5^ Similar situations have been encountered with mechanistically different enzymes, including triosephosphate isomerase,^28^ orotidine 5′-monophosphate decarboxylase,^29^ and 1-deoxy-D-xylulose-5-phosphate reductoisomerase,^30^ where interactions with the PO_4_^2–^ group enable up to 13 kcal/mol stabilization of the transition state.^31^ Interestingly, no significant differences in active site conformation are seen between the *Hs*DNPH1^Trunc^:dUMP complex and *Hs*DNPH1 bound to 6-naphthyl-AMP (Figure S5B), a ribonucleotide analogue that inhibits the enzyme.^8^ The −COO^–^ groups of Glu104 and the conserved Asp80^25^ are on average ∼5.4 Å apart (Figure 2C), reminiscent of the distance between the nucleophilic and general acid −COO^–^ groups of retaining glycoside hydrolases whose reactions commonly proceed via covalent catalysis.^32^

### *Hs*DNPH1-Catalyzed Hydrolysis of dUMP

**Figure 3:**
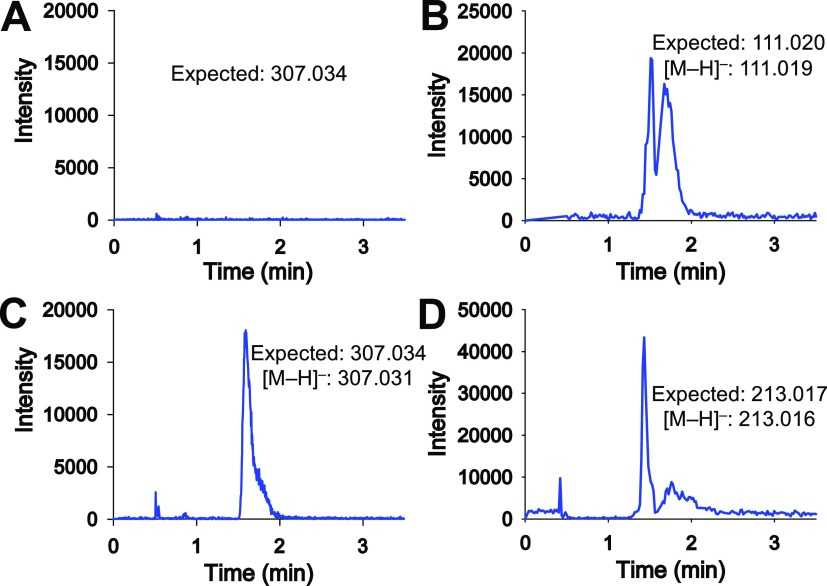
LC-ESI-MS analysis of *Hs*DNPH1-catalyzed hydrolysis of dUMP. (A) Elution profile of extracted ion of mass corresponding to dUMP after hydrolysis by *Hs*DNPH1. (B) Elution profile of the extracted ion of mass corresponding to uracil after dUMP hydrolysis by *Hs*DNPH1. The peak on the left is likely an impurity from the dUMP with the same mass as uracil but a retention time of 1.58 min as opposed to uracil’s 1.66 min. (C) Elution profile of the extracted ion of mass corresponding to dUMP in a control reaction lacking *Hs*DNPH1. (D) Elution profile of the extracted ion of mass corresponding to 2-deoxyribose 5-phosphate after dUMP hydrolysis by *Hs*DNPH1.

To verify the formation of uracil and 2-deoxyribose 5-phosphate upon *Hs*DNPH1-catalyzed cleavage of the *N*-ribosidic bond of dUMP, the reaction was analyzed by high-resolution LC-ESI-MS (Figure 3). In the control mixture lacking enzyme, only ions corresponding to the dUMP mass ([M – H]^−^: 307.031) were detected after 2 h. In the reaction mixture, no dUMP could be detected after 2 h, in agreement with the expected irreversibility of the hydrolytic reaction, and ions corresponding to the masses of uracil ([M – H]^−^: 111.019) and 2-deoxyribose 5-phosphate ([M – H]^−^: 213.016) were readily detected. This confirms dUMP as a substrate for *Hs*DNPH1 as previously reported,^6^ likely a slow-reacting one in comparison with the physiological 5hmdUMP.^1^*Hs*DNPH1 and *Hs*DNPH1^Trunc^ catalyze dUMP hydrolysis with comparable rates (Figure S6), suggesting that *Hs*DNPH1^Trunc^ is catalytically active, and *in-crystallo* trapping of dUMP is probably due to inherently slow reaction rates with this substrate.

### Acid–Base Chemistry

**Figure 4:**
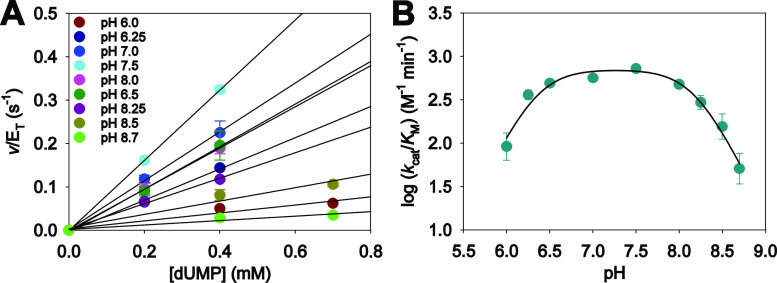
*Hs*DNPH1 pH-rate profile. (A) Initial rates of *Hs*DNPH1-catalyzed hydrolysis of dUMP at different pH values. Data points are mean ± SEM, and lines are best fit to eq 1. (B) *Hs*DNPH1 *k*_cat_/*K*_M_ profile for dUMP hydrolysis. Data points are mean ± fitting error. The line is best fit to eq 2.

To probe the role of acid–base chemistry in *Hs*DNPH1 substrate binding and/or catalysis, *k*_cat_/*K*_M_ for dUMP hydrolysis was obtained at various pHs upon best fit of initial-rate data to eq 1 (Figure 4A), resulting in a bell-shaped pH-rate profile (Figure 4B). Best fit of the data to eq 2 indicated that 2 groups with a p*K*_a_ of 6.4 ± 0.2 must be deprotonated for maximum *k*_cat_/*K*_M_, while 2 groups with a p*K*_a_ of 8.2 ± 0.1 must be protonated. Under the assumption that the apparent LBHB is important for binding and/or catalysis, it is possible to hypothesize that if Glu104 were protonated below pH 6.4, the apparent LBHB would not form, as would be the case if Tyr24 were deprotonated above pH 8.2, leading to abolition of catalysis. A condition for an LBHB is that the groups transiently sharing hydrogen must have similar p*K*_a_.^26^ Therefore, Glu104 and Tyr24 cannot have p*K*_a_s of 6.4 and 8.2, respectively, while participating in an apparent LBHB. However, the hypothesis outlined above could be operational provided that at any point in the catalytic cycle from free enzyme and free substrate up to and including the first irreversible step, the apparent LBHB is absent long enough for Glu104 and Tyr24 to undergo equilibrium proton transfer.

A candidate for the second group with a p*K*_a_ of 8.2 that must be protonated for maximum *k*_cat_/*K*_M_ is Asp80 as a negatively charged −COO^–^ group would electrostatically destabilize departure of the leaving group at the transition state, which is likely the anionic form of uracil as evidenced for other enzyme-catalyzed *N*-ribosidic bond cleavage reactions of pyrimidine nucleosides and nucleotides.^33−36^ Increased p*K*_a_s, even higher than 8.2, for carboxylic acid side chains have been reported to be operational in enzymes,^37,38^ particularly if the side chain is near other potentially negatively charged residues and/or in a hydrophobic microenvironment.^38^ For instance, the general acid/general base Glu residue in a mutant retaining xylanase from *Bacillus circulans* has a p*K*_a_ of 8.4 as determined by ^13^C NMR titration.^39^ Asp80 is ∼5 Å from Glu104 and surrounded by Ile76, Trp83, and Met130. This scenario is even compatible with Asp80 serving as a general acid to protonate the leaving group after *N*-ribosidic bond cleavage to generate the neutral uracil product. Another candidate might be Cys26 as 8.2 is in the range of −SH group p*K*_a_s in proteins.^38,40^ As the Cys26 −SH group lies ∼3.7 Å from the Glu104 −COO^–^ group, deprotonation of the thiol could elevate the Glu104 side chain p*K*_a_, decreasing its nucleophilicity.

The p*K*_a_ of 6.4 matches the second p*K*_a_ of the 5′-PO_4_^2–^ group of dUMP, so this could be the second group that must be deprotonated for maximum *k*_cat_/*K*_M_. The 5′-PO_4_^2–^ group is essential for binding as 2′-deoxynucleosides are not substrates of DNPH1.^5^ Protonation of dUMP 5′-PO_4_^2–^ group may destabilize one or more H-bonds with the Ser residues it interacts with. An analogous situation was reported for dUMP interaction with thymidylate synthase, where MD simulations indicated that protonation of one of the 5′-PO_4_^2–^ oxygens increased binding energy significantly.^41^

### Solvent Viscosity Effects

**Figure 5:**
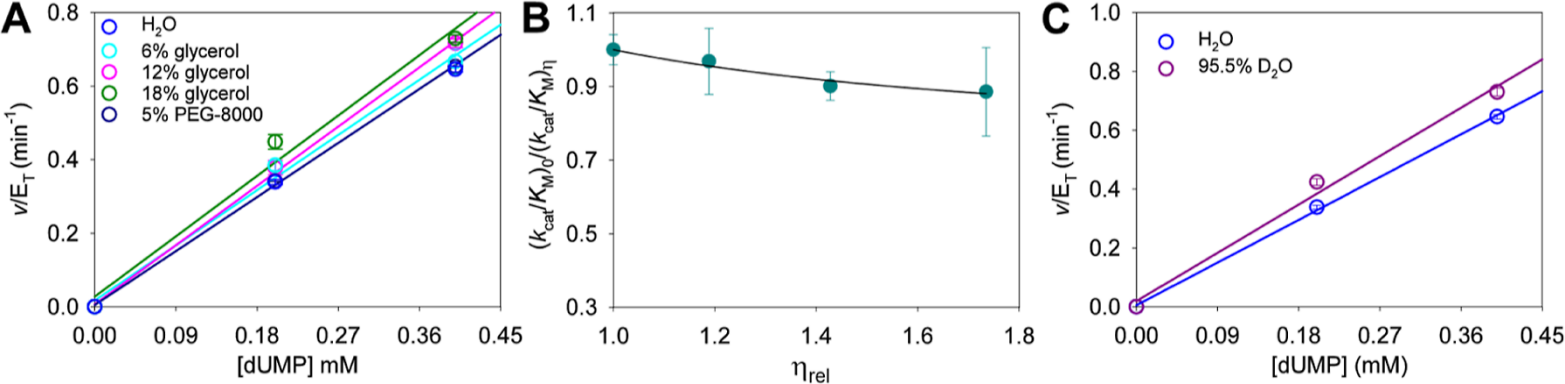
Solvent effects on *Hs*DNPH1-catalyzed dUMP hydrolysis. (A) Initial rates at low dUMP concentrations in different medium viscosities. Data points are mean ± SD, and lines are best fit to eq 1. (B) Solvent viscosity effects on *k*_cat_/*K*_M_. Data points are mean ± propagated fitting error, and the line is best fit to eq 3. Parameters *A* and *B* in eq 3 were associated with large fitting errors, probably due to the plateau region not being fully reached in the graph. However, this does not affect the conclusions drawn from the inverse solvent viscosity effect. (C) Initial rates at low dUMP concentrations in H_2_O or 95.5% D_2_O (v/v). The data in H_2_O are the same as in (A). Data points are mean ± SD, and lines are best fit to eq 1.

To gather information on whether diffusional steps contribute to *Hs*DNPH1 *k*_cat_/*K*_M_, solvent viscosity effects were determined by measuring initial rates at low dUMP concentrations at different concentrations of the microviscogen glycerol (Figure 5A, Table S2). The *k*_cat_/*K*_M_ in the absence of glycerol was 1615 ± 47 M^–1^ min^–1^, in very good agreement with the reported value of 1538 M^–1^ min^–1^ based on dUMP saturation curves in an HPLC-based assay.^6^ A plot of *k*_cat_/*K*_M_ ratios against relative viscosity showed a modestly inverse viscosity effect (Figure 5B), ruling out diffusional steps as rate limiting for *Hs*DNPH1 *k*_cat_/*K*_M_ (which includes binding of dUMP and release of uracil)^22^ but suggesting a form of enzyme with higher binding and/or catalytic efficiency is favored in a more viscous medium.^22,42^ As expected, *Hs*DNPH1 rates were insensitive to the macroviscogen PEG-8000 (Figure 5A).

As the rat DNPH1 can catalyze the methanolysis of 2′-deoxyguanosine 5′-monophosphate,^10^ the possibility of *Hs*DNPH1-catalyzed transfer of 2-deoxyribose 5-phosphate to glycerol was considered and tested using a ^1^H NMR comparative analysis (Figure S7). No noticeable difference is seen in the spectra for reactions carried out in the presence or absence of 18% glycerol. Given the low catalytic rate of *Hs*DNPH1 with dUMP, formation of low concentrations of a glycerol-2′-deoxyribose 5′-phosphate adduct cannot be ruled out, but it does not seem to be a significant reaction trajectory as compared to hydrolysis of dUMP. It should be pointed out that even if reaction trajectories leading to glycerol-2′-deoxyribose 5′-phosphate happened to a significant extent, they would not contribute to *k*_cat_/*K*_M_ and would not affect the solvent viscosity effects reported above.

### Solvent Deuterium Isotope Effects

To uncover possible rate-limiting proton transfers in the *Hs*DNPH1-catalyzed dUMP hydrolysis, solvent deuterium isotope effects were measured at low dUMP concentrations (Figure 5C). Based on the ratio of slopes of the relevant curves, an apparent  of 0.88 ± 0.04 was obtained, a distinct value from the solvent viscosity effect of 0.97 ± 0.07 at 6% glycerol, which is approximately equivalent to the viscosity of 95.5% D_2_O at 37 °C.^43,44^ Interestingly, this  is similar to those measured for HIV-1 protease (0.85) by Rodriguez and Meek as reported by Northrop^45^ and for porcine pepsin (0.84).^46^ The inverse  for HIV-1 protease and porcine pepsin has been attributed to an LBHB involving the catalytic Asp residues of these aspartic proteases.^45^ Proton transfer from an LBHB will produce an inverse solvent equilibrium deuterium isotope effect  if the proton acceptor has a fractionation factor near unity and the transfer precedes a large energy barrier since LBHBs have inverse fractionation factors.^47^

**Figure 6:**
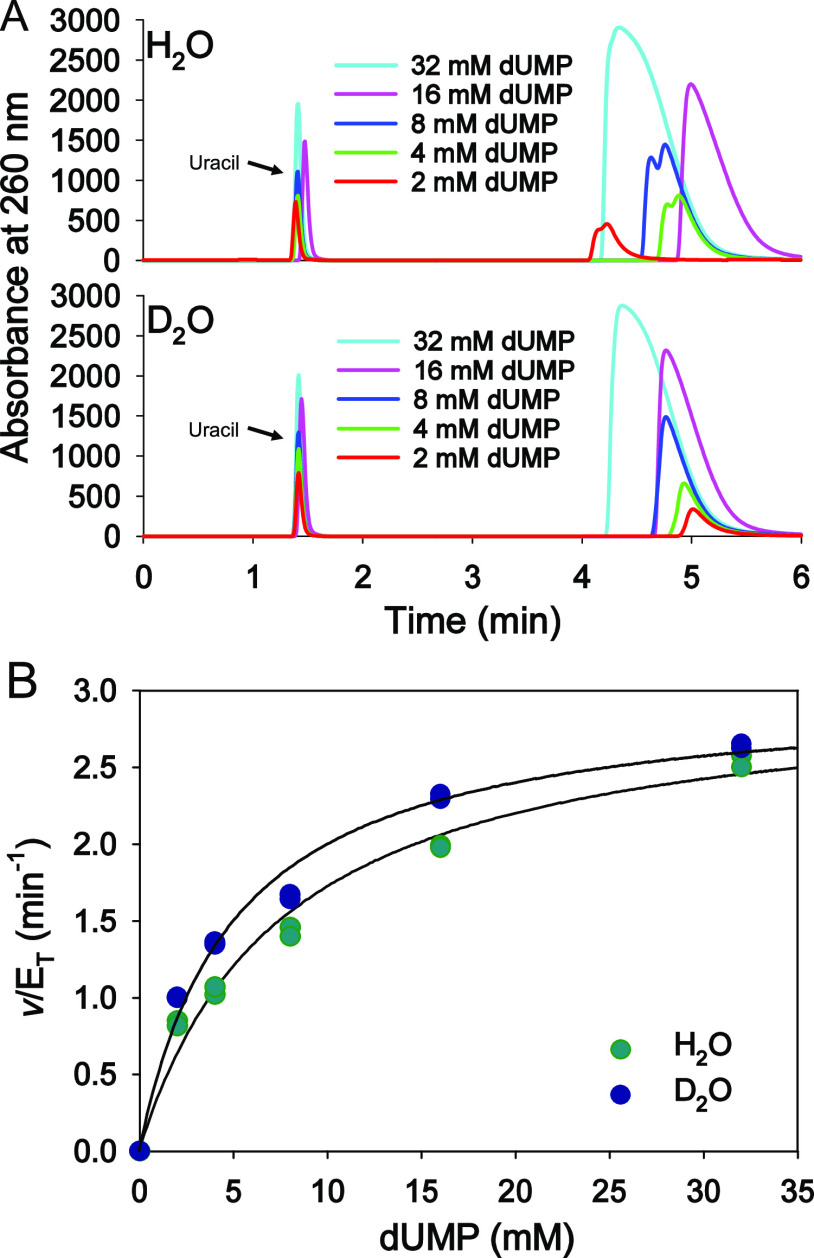
Solvent deuterium isotope effects. (A) Representative chromatograms of *Hs*DNPH1-catalyzed dUMP cleavage in H_2_O and D_2_O. Two independent experiments were carried out in H_2_O and D_2_O. (B) *Hs*DNPH1 saturation curves with dUMP in H_2_O and D_2_O. All data points are shown. Lines are best fit to either eq 4 (H_2_O curve) or eq 5 (D_2_O curve).

In dUMP hydrolysis catalyzed by *Hs*DNPH1, the first irreversible step is arguably the dissociation of uracil from the enzyme, which presumably precedes hydrolysis of the putative 5-phospho-2-deoxyribosyl-enzyme intermediate, precluding information on the second half-reaction to be gleaned from . To assess if a proton transfer might be rate limiting in the overall reaction, initial rates of dUMP cleavage were measured in H_2_O and 90% D_2_O via an HPLC-based assay (Figures 6A, S8, and S9) to obtain dUMP saturation curves (Figure 6B). Fitting the data in H_2_O to eq 4 yielded a *K*_M_ of 8 ± 1 mM, in excellent agreement with that previously reported,^6^ and a *k*_cat_ of 3.0 ± 0.2 min^–1^, fourfold lower than a previously published value.^6^ For comparison, a *K*_M_ of 6.3 μM and a *k*_cat_ of 19.8 min^–1^ have been recently reported for *Hs*DNPH1-catalyzed hydrolysis of the physiological substrate 5hmdUMP at 37 °C.^48^ The data in D_2_O were best fitted to eq 5, which excludes an isotope effect on , resulting in a  of 0.62 ± 0.07. Fitting eq 4 to the data in D_2_O and calculating isotope effects by taking the ratio of the rate constants in H_2_O to the respective rate constants in D_2_O produced a  of 1.0 ± 0.1 and a  of 0.7 ± 0.1. These  values are similar, albeit not identical to the  measured in the spectrophotometric assay, possibly due to the inherently higher experimental error of the discontinuous assay in comparison to a direct and continuous one. The unity  rules out a rate-limiting proton-transfer step from the *Hs*DNPH1:dUMP complex to release of 2-deoxyribose 5-phosphate.^49^

### Proposed Catalytic Mechanism

**Scheme 2:**
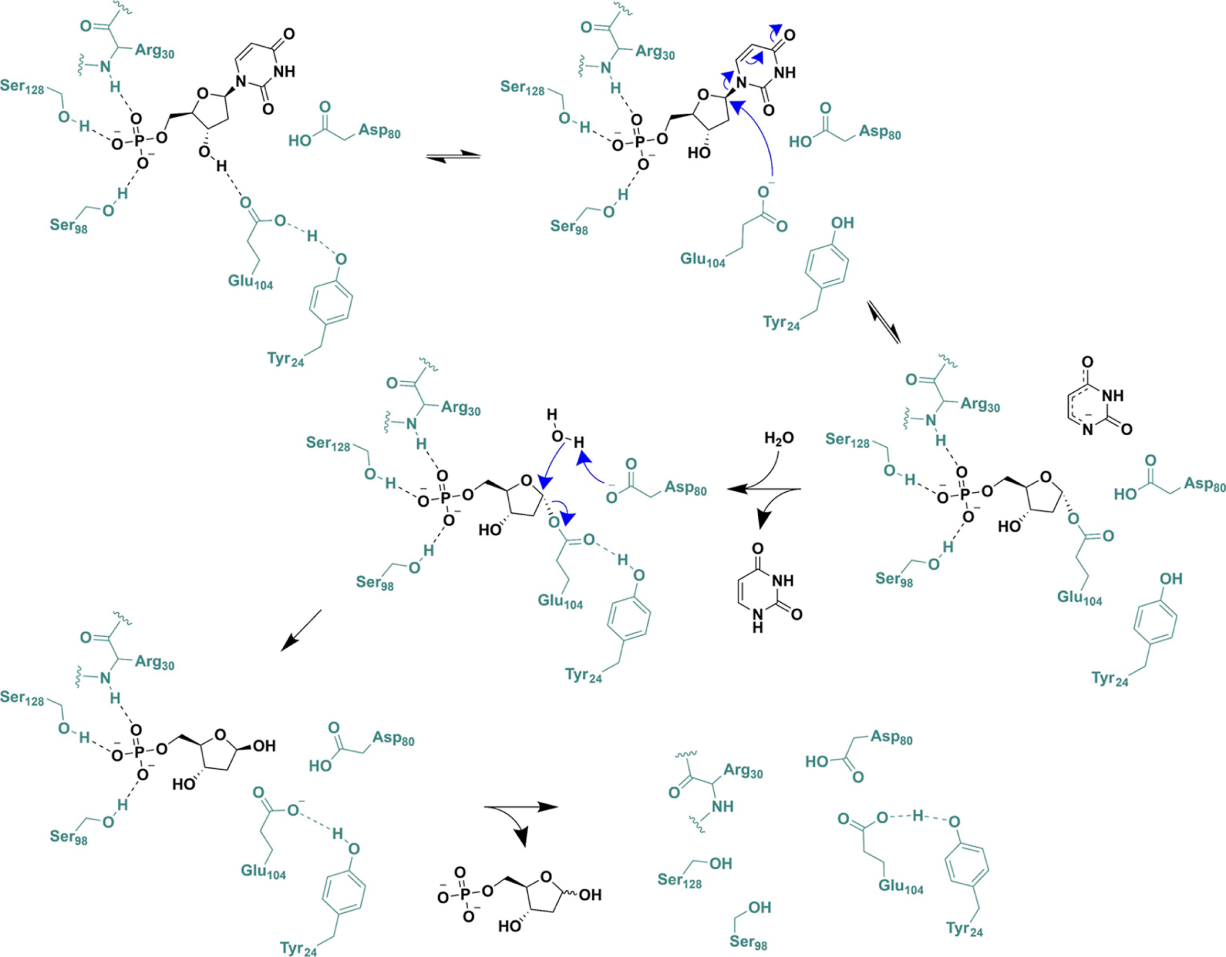
Proposed Mechanism for *Hs*DNPH1-Catalyzed Hydrolysis of dUMP

*Hs*DNPH1 is a promising target for inhibition to potentiate PARPi and overcome resistance to BRCA-deficient cancer chemotherapy.^1^ A detailed description of the *Hs*DNPH1 mechanism may help inform the design of potent and specific inhibitors. Integrating the structural and kinetic data presented in this study, along with previously published data for the rat orthologue of DNPH1 and the precedent furnished by nucleoside 2′-deoxyribosyltransferases and glycoside hydrolases, a double-displacement mechanism for dUMP hydrolysis catalyzed by *Hs*DNPH1 is proposed (Scheme 2).

An apparent LBHB between Glu104 and Tyr24 is present in the unbound and dUMP-bound crystal structures. While its presence must yet be confirmed and its role and importance for the catalytic reaction remain to be elucidated, p*K*_a_s of essential groups in binding and/or catalysis are compatible with deprotonated Glu104 and protonated Tyr24 being required to establish the apparent LBHB. The reaction is hypothesized to proceed via covalent catalysis based on the similarity of the *Hs*DNPH1 active site with that of nucleoside 2′-deoxyribosyltransferases,^5^ the catalytic essentiality of Glu104,^1^ and the retention of configuration on the anomeric carbon upon 2′-deoxynucleoside 5′-phosphate methanolysis catalyzed by the rat DNPH1.^10^ Glu104, Tyr24, and Asp80 are highly conserved across DNPH1 orthologues,^25^ and mutation of the equivalent Tyr residue for a Phe residue in the rat DNPH1 caused a 280-fold reduction in *k*_cat_/*K*_M_.^10^ Interestingly, the distance between the Glu104 and Asp80 −COO^–^ groups in *Hs*DNPH1 is ∼5.4 Å (Figure 2C). The plethora of structural data on glycoside hydrolases demonstrate that the distance between the two catalytically important −COO^–^ groups correlates strongly with their mechanism: a distance of ∼5 Å is a hallmark of retaining glycoside hydrolases whose catalytic mechanism involves a glycosylated Glu residue, while a distance of ∼10 Å is characteristic of inverting glycoside hydrolases operating via direct water attack.^32^

The apparent LBHB presumably must be broken to allow nucleophilic attack of Glu104 on the anomeric carbon as it would otherwise reduce the nucleophilicity of the −COO^–^ group. This process would give rise to an inverse  as observed here, provided no other rate-limiting proton transfer takes place in the steps encompassed by *k*_cat_/*K*_M_.^47^ The possibility exists that the  of 0.88 reflects an inverse  on the breaking of the apparent LBHB. The small increase in the apparent LBHB length in the Michaelis complex as compared with the unliganded enzyme, still insufficient to disrupt the apparent LBHB, might reflect the poor substrate nature of the dUMP (*k*_cat_/*K*_M_ of 27 M^–1^ s^–1^). It could be envisioned that binding of the physiological substrate 5hmdUMP (*k*_cat_/*K*_M_ of 52 380 M^–1^ s^–1^)^48^ would more efficiently disrupt the apparent LBHB to promote nucleophilic attack by Glu104. A neutral Asp80 side chain would avoid destabilization of the anionic uracil at the transition state and would serve as the general acid to establish the neutral uracil form after the *N*-hydrolytic bond is severed. Protonation of the anionic form of uracil and/or departure of the neutral product from the enzyme represent the first irreversible step, considering the relatively high p*K*_a_ of uracil (9.5).^50,51^ Asp80 then deprotonates a water molecule to facilitate nucleophilic attack on C1 of the 5-phospho-2-deoxyribosyl-enzyme intermediate to generate 2-deoxyribose 5-phosphate which departs the enzyme. This would probably require the p*K*_a_ of Asp80 to be lowered significantly in the 5-phospho-2-deoxyribosyl-enzyme intermediate from its value in the free enzyme. Precedence for this is found, for example, in a *B. circulans* retaining xylanase, where a Glu residue acts first as a general acid to protonate the leaving group, followed by a decrease of ∼2.5 units in its p*K*_a_ in the covalent enzyme intermediate to act as a general base to activate water as a nucleophile.^52^ In *Hs*DNPH1, the solvent deuterium isotope effects indicate that any proton-transfer steps, including activation of water, are fast relative to other steps. This work will help pave the way for the generation and functional characterization of *Hs*DNPH1 active-site mutants to elucidate the role of specific residues and the apparent LBHB in catalysis.

## Supporting Information Available

Further structural, biophysical, and biochemical results and analysis of *Hs*DNPH1 and its reaction (PDF)

## Accession Codes

*Hs*DNPH1: UniProt O43598. PDB ID: 8OS9 and 8OSC.

## Abbreviations

DNPH1: 2′-deoxynucleoside 5′-phosphate *N*-hydrolase 1
5hmdUMP: 5-hydroxymethyl-2′-deoxyuridine 5′-monophosphate
BRCA: breast cancer gene
PARPi: poly(ADP-ribose) polymerase inhibitors
*Hs*DNPH1: *Homo sapiens* DNPH1
dUMP: 2′-deoxyuridine 5′-monophosphate
LC-ESI-MS: liquid chromatography-electrospray ionization-mass spectrometry
DTT: dithiothreitol
IPTG: isopropyl-β-D-1-thiogalactopyranoside
MES: 2-(*N*-morpholino)ethanesulfonic acid
Tris: tris(hydroxymethyl)aminomethane
TAPS: *N*-[tris(hydroxymethyl)methyl]-3-aminopropanesulfonic acid
HEPES: 4-(2-hydroxyethyl)piperazine-1-ethanesulfonic acid
PEG-8000: polyethylene glycol 8000
TEVP: tobacco etch virus protease
*k*_cat_: steady-state catalytic constant
H-bond: hydrogen bond
LBHB: low-barrier hydrogen bond
