# Supplementary material for: Human 2′-Deoxynucleoside 5′-Phosphate N-Hydrolase 1: Mechanism of 2′-Deoxyuridine 5′-Monophosphate Hydrolysis
Source: Biochemistry. 2023 Aug 15;62(17):2658–68. doi: 10.1021/acs.biochem.3c00369 (PMC10483697; doi:10.1021/acs.biochem.3c00369)
Supplement: Supplementary file 1 — bi3c00369_si_001.pdf [file bi3c00369_si_001.pdf]

Supporting Information

Human 2′-deoxynucleoside 5′-phosphate *N*-hydrolase 1: mechanism of 2′-deoxyuridine 5′-monophosphate hydrolysis

Suneeta Devi,[†] Anna E. Carberry,[†] Greice M. Zickuhr,[§] Alison L. Dickson,[§,‡] David J. Harrison,[§,‡] and Rafael G. da Silva[†,*]

[†]School of Biology, Biomedical Sciences Research Complex, University of St Andrews, St Andrews, KY16 9ST, United Kingdom

[§]School of Medicine, University of St Andrews, St Andrews, KY16 9TF, United Kingdom

[‡]NuCana Plc, Edinburgh, EH12 9DT, United Kingdom

*To whom correspondence may be addressed: rgds@st-andrews.ac.uk, phone: +44 01334 463496

RESULTS

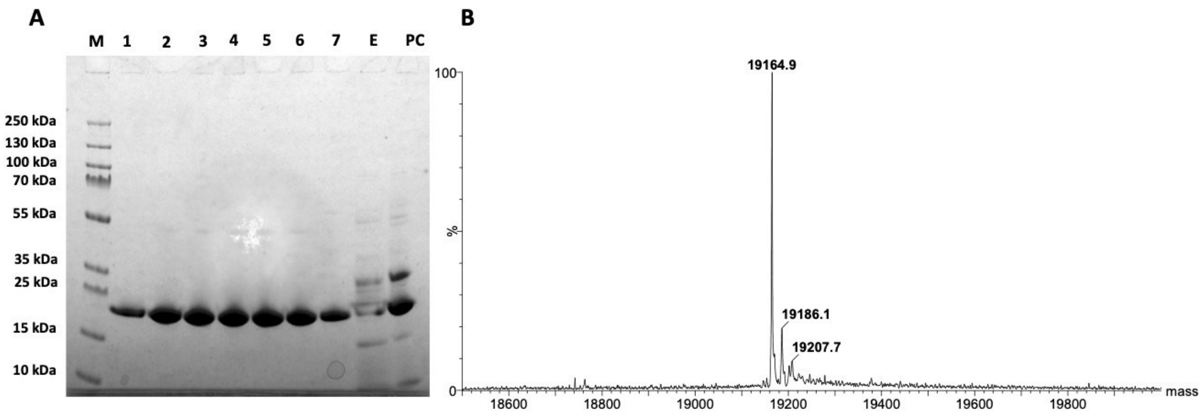

Figure S1. *Hs*DNPH1 purification. (A) Coomassie Blue-stained SDS-polyacrylamide gel of purified *Hs*DNPH1 after the second affinity chromatography after cleavage with TEVP. Lane M denotes PageRuler Plus Prestained Protein Ladder, the molecular weight marker. Lane PC, the sample that was loaded onto the column. Lane E is the eluate in the imidazole gradient. Lanes 1 – 7, the fractions pooled from the flow through. (B) ESI-MS analysis of purified *Hs*DNPH1.

***Hs*DNPH1**	GMAAAMVPGRSESWERGEPGRPALYFCGSIRGGREDRTLYERIVSRLRRFGTVLTEHVAA
***Hs*DNPH1^{Trunc}**	GMRPALYFCGSIRGGREDRTLYERIVSRLRRFGTVLTEHVAA
***Hs*DNPH1**	AELGARGEEAAGGDRLIHEQDLEWLQQADVVVAEVTQPSLGVGYELGRAVAFNKRILCLF
***Hs*DNPH1^{Trunc}**	AELGARGEEAAGGDRLIHEQDLEWLQQADVVVAEVTQPSLGVGYELGRAVAFNKRILCLF
***Hs*DNPH1**	RPQSGRVLSAMIRGAADGSRFQVWDYEEGEVEALLDRYFEADPPGQVAASPDPTT
***Hs*DNPH1^{Trunc}**	RPQSGRVLSAMIRGAADGSRFQVWDYEEGEVEALLDRYFEADP

Figure S2. Amino acid sequence of full-length *Hs*DPNH1 (blue) and truncated *Hs*DNPH1 (black) produced in this study. The N-terminus of each protein has a Gly before the Met residue, left over from TEVP cleavage.

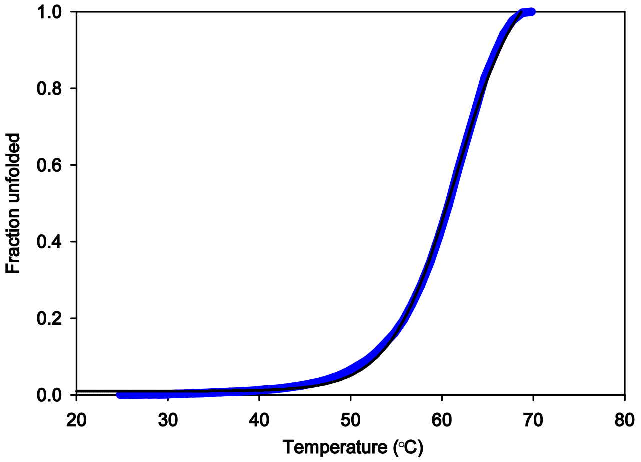

Figure S3. DSF-based thermal denaturation of *Hs*DPNH1. The black line is the best fit to the following equation: $F_U = LL + \frac{UL-LL}{1+e^{(Tm-T)/c}}$, where F_U is fraction unfolded, T is the temperature in °C, T_m is the melting temperature, c is the slope of the transition region, and LL and UL are folded and unfolded baselines, respectively.[1]

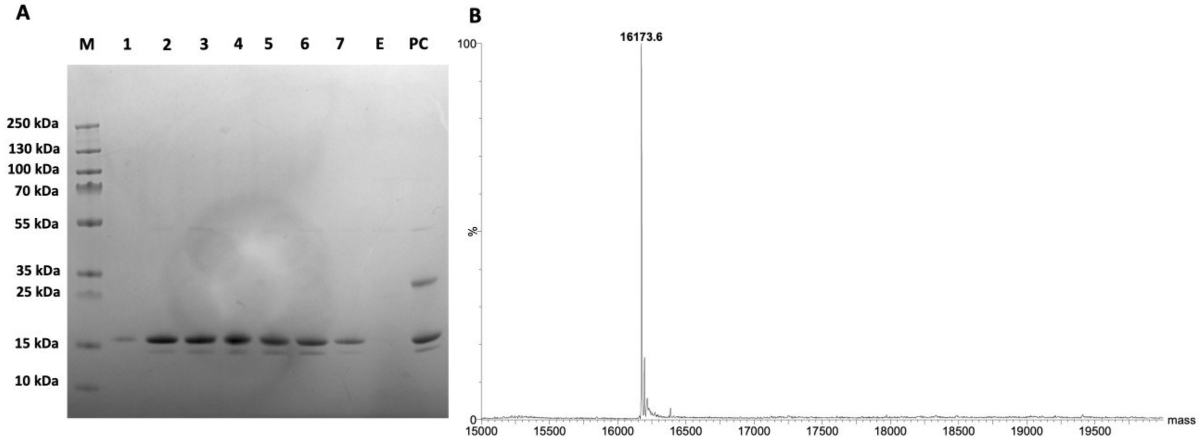

Figure S4. *Hs*DNPH1^{Trunc} purification. (A) Coomassie Blue-stained SDS-polyacrylamide gel of purified *Hs*DNPH1^{Trunc} after the second affinity chromatography after cleavage with TEVP. Lane M denotes PageRuler Plus Prestained Protein Ladder, the molecular weight marker. Lane PC, the sample that was loaded onto the column. Lane E is the eluate in the imidazole gradient with. Lanes 1 – 7, the fractions pooled from the flow through. (B) ESI-MS analysis of purified *Hs*DNPH1^{Trunc}.

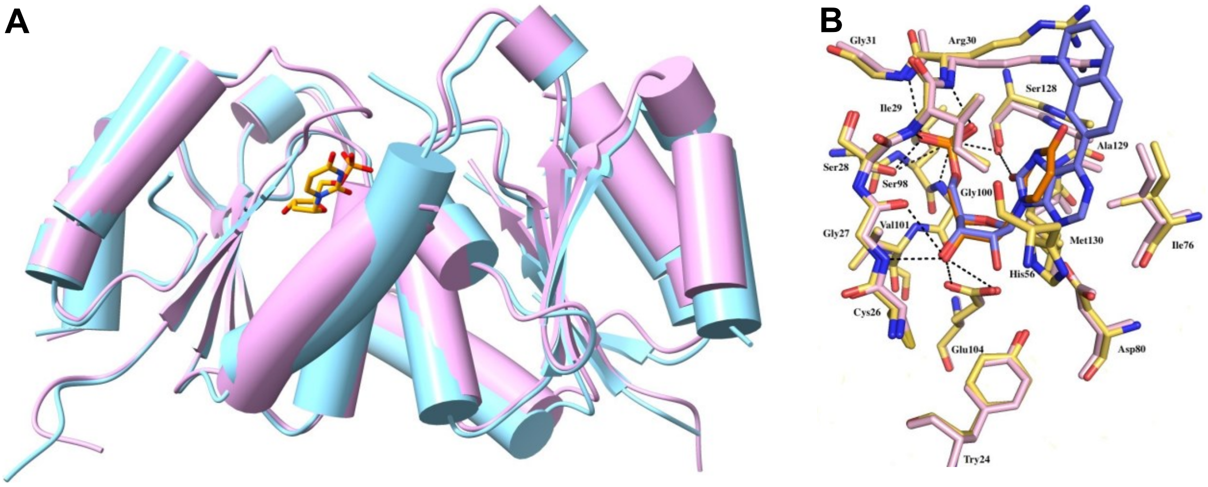

Figure S5. *Hs*DNPH1 structural overlay. (A) Overlay between unbound *Hs*DNPH1^{Trunc} (blue) and dUMP-bound complex (pink) crystal structures. (B) Active-site close-up of the overlaid structures of dUMP-bound *Hs*DNPH1^{Trunc} (salmon) and 6-naphthyl-AMP-bound *Hs*DNPH1 (yellow) (PDB ID: 4P5E).

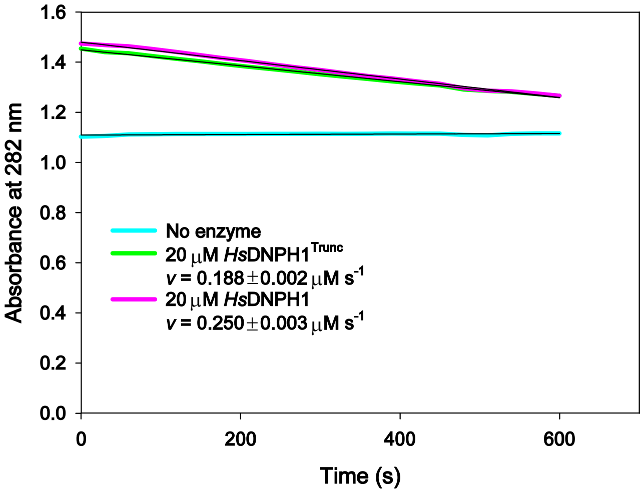

Figure S6. Rates of hydrolysis of dUMP catalysed by either *Hs*DNPH1 or *Hs*DNPH1^{Trunc}. Thick lines are average of two independent traces. Thin black lines are linear regressions of the data. Rates are shown as mean ± fitting error. In all cases, the initial concentration of dUMP was 400 μM.

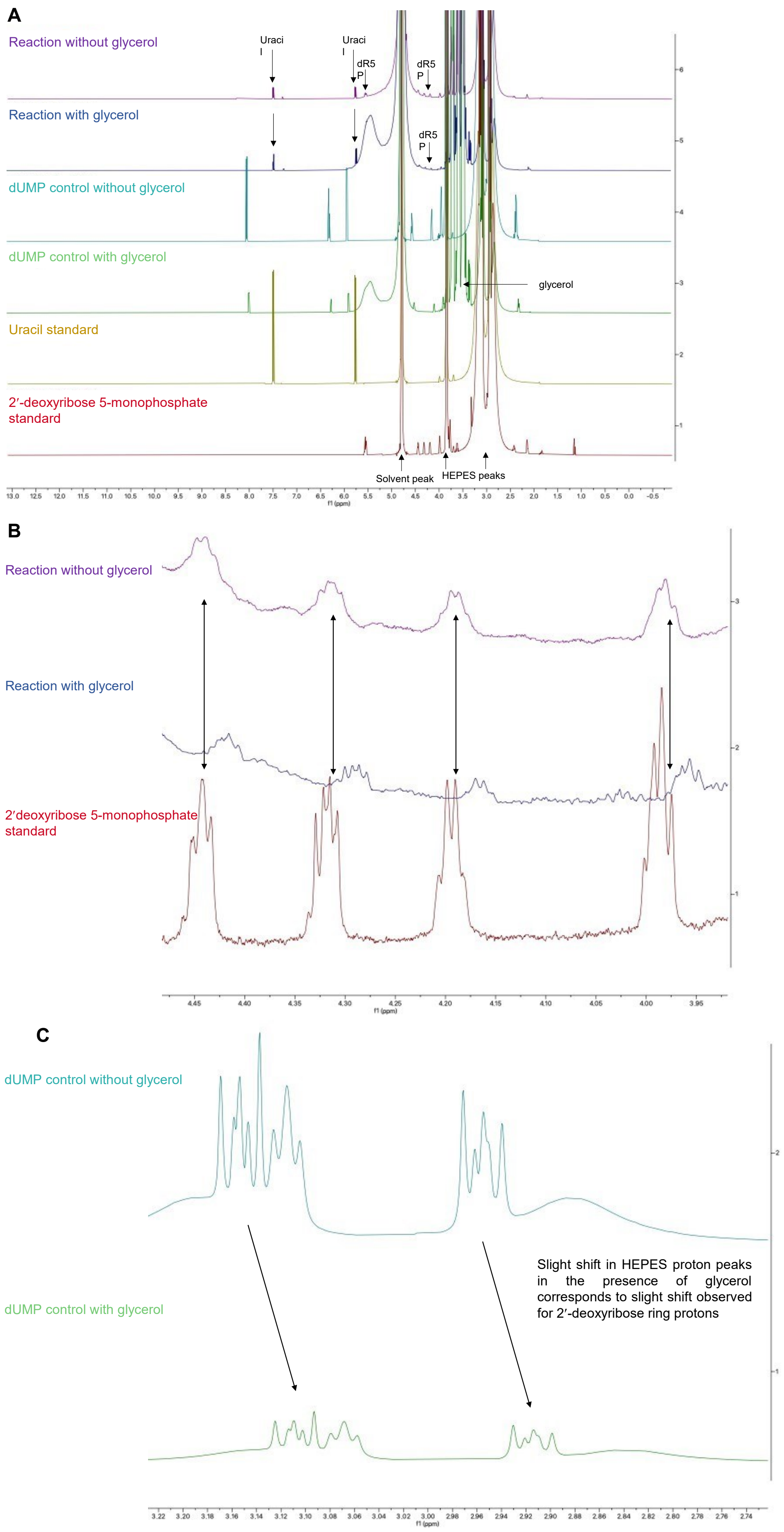
A
Reaction without glycerol
Reaction with glycerol
dUMP control without glycerol
dUMP control with glycerol
Uracil standard
2′-deoxyribose 5-monophosphate standard
Uracil
dR5P
glycerol
Solvent peak
HEPES peaks
f1 (ppm)
B
Reaction without glycerol
Reaction with glycerol
2′deoxyribose 5-monophosphate standard
f1 (ppm)
C
dUMP control without glycerol
dUMP control with glycerol
Slight shift in HEPES proton peaks in the presence of glycerol corresponds to slight shift observed for 2′-deoxyribose ring protons
f1 (ppm)

Figure S7. ^{1}H-NMR spectra. (A) Stacking of the spectra for standards, controls without HsDNPH1 (with and without 18% glycerol), and reactions (with and without 18% glycerol). Key regions in the spectra are indicated by arrows to corresponding to protons of different compounds. (B) Close-up view of the region corresponding to 2′-deoxyribose 5-phosphate protons. (C) Close-up view of the region corresponding to HEPES protons, indicating a slight up-field shift brought about by the presence of glycerol, which is also noted in other regions. All spectra were aligned by the solvent peak.

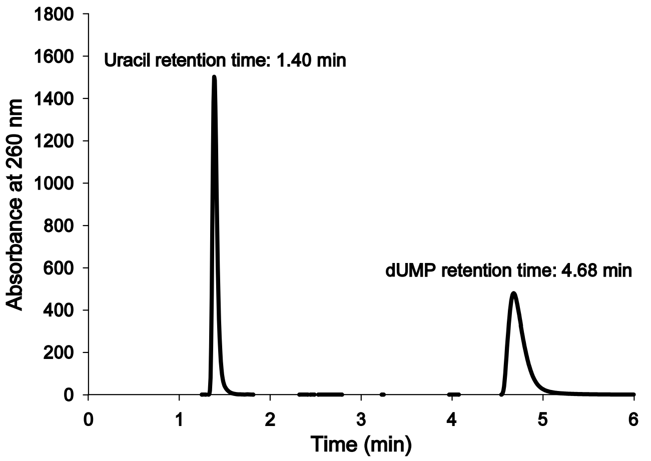

Figure S8. HPLC chromatogram of a solution of uracil and dUMP. The identity of the uracil peak was confirmed based on the uracil retention time in the standard curve.

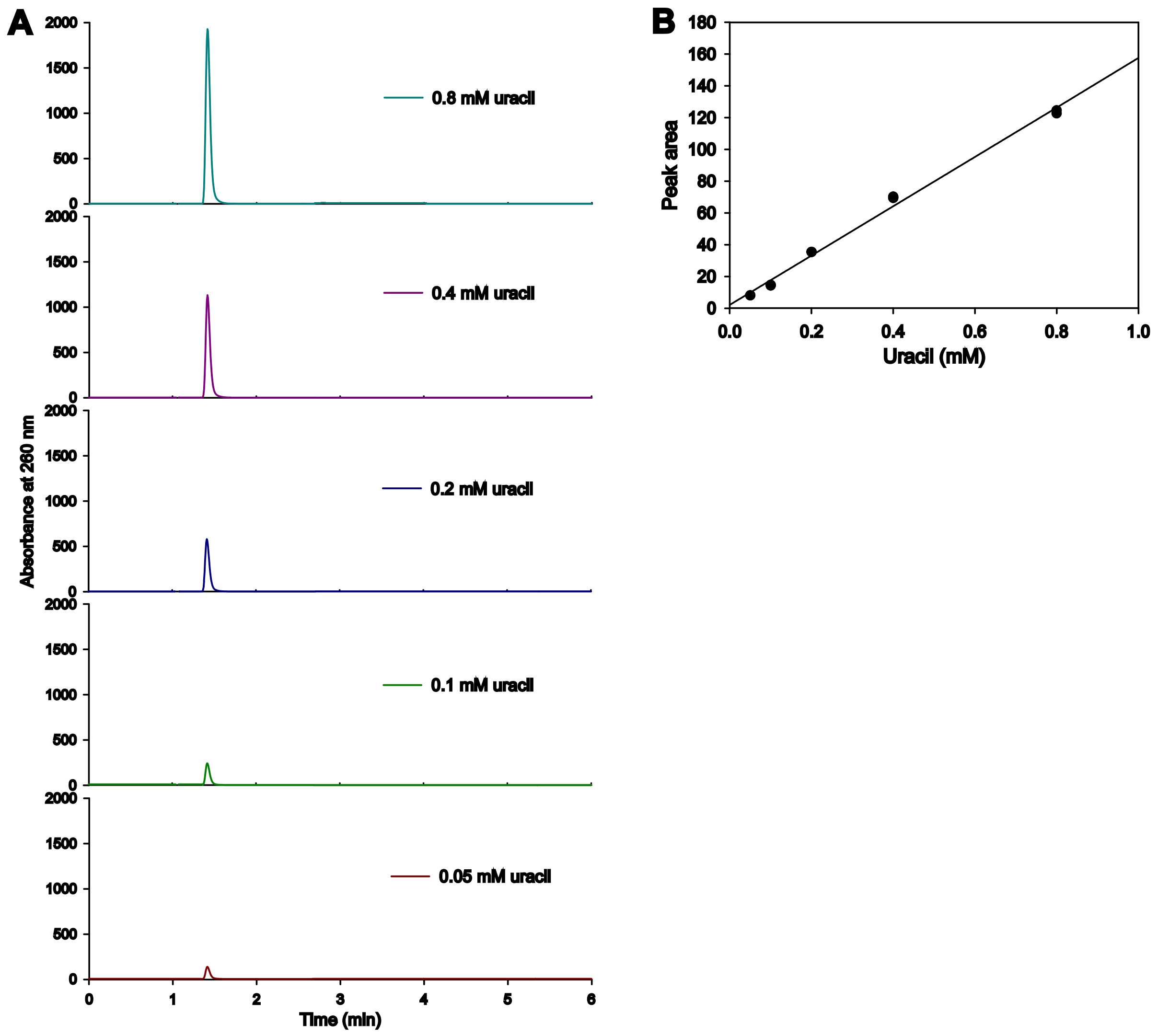

Figure S9. HPLC standard curve for uracil. (A) Representative chromatograms of one experiment of a total of two. Each uracil peak was integrated. (B) Standard curve for uracil. All data points are plotted. The line is a linear regression of the data (R^2 = 0.997), producing slope of 155 mM^{-1} and y-axis intercept of 2.2.

Table S1. Data processing and refinement statistics of unbound *Hs*DNPH1^{Trunc} and dUMP-bound crystal structures.[a]

	**Unbound *Hs*DNPH1^{Trunc}**	**dUMP-bound *Hs*DNPH1^{Trunc}**
**Data Collection**		
**Beamline**	I04, DLS	I04, DLS
**Wavelength (Å)**	0.9537	0.9537
**Space group**	$P1\ 2_1\ 1$	$C1\ 2\ 1$
**a, b, c (Å)**	57.49, 64.51, 62.93	107.33, 38.76, 66.20
**α, β, γ (°)**	90, 90.03, 90	90, 120.29, 90
**Resolution range (Å)**	35.47 – 1.7 (1.761 – 1.7)	33.38 – 1.42 (1.44 – 1.42)
**Total reflections (unique reflections)**	310685 (45873)	685731 (44559)
**Completeness (%)**	90.4 (99.5)	99.7 (94.9)
**Multiplicity**	6.8 (6.2)	15.4 (10.9)
**Mean I/sigma(I)**	18.3 (2.2)	24.7 (4.0)
**Wilson B-factor**	30.42	19.73
**R-merge**	0.44 (0.609)	0.058(0.814)
**$CC_{1/2}$**	0.99 (0.85)	0.99 (0.89)
**Refinement**		
**R_{work}/R_{free}**	0.2074/0.226	0.1985/0.2077
**Number of atoms**	4071	2172
**Protein**	3907	1960
**Ligands**	0	20
**Water**	164	192

**Protein residues**	484	244
**RMSD**		
**Bond length (Å)**	0.009	0.010
**Bond angles (º)**	1.15	1.23
**Ramachandran Plot (%)**		
**Favoured**	98.70	98.72
**Allowed**	1.30	1.28
**Outliers**	0.00	0.00
**Average B-factor (Å^2)**	38.88	24.67
**Macromolecules**	38.73	23.61
**Solvent**	42.48	34.66
**Ligands**	N/A	32.58
**PDB ID**	8OS9	8OSC

[a]Data in brackets are for the highest resolution shell.

Table S2. *Hs*DNPH1 k_{cat}/K_M in different reaction media.

**Reaction medium**	**k_{cat}/K_M (M^{-1} min^{-1})**
H_2O	1615 ± 47
6% glycerol	1666 ± 147
12% glycerol	1792 ± 57
18% glycerol	1823 ± 242
5% PEG-8000	1632 ± 42
D_2O	1825 ± 76

REFERENCES

(1) Niesen, F. H., Berglund, H., and Vedadi, M. The use of differential scanning fluorimetry to detect ligand interactions that promote protein stability. *Nat. Protoc.* **2007**, *2*, 2212-2221.
